# Age-Related Differences in the Functional Demand Placed on the Lumbar Spine during Walking in Healthy Older versus Younger Men

**DOI:** 10.3390/geriatrics9050108

**Published:** 2024-08-23

**Authors:** Alexander Dallaway, Michael Duncan, Corbin Griffen, Derek Renshaw, Jason Tallis, John Hattersley

**Affiliations:** 1 School of Health and Society, Faculty of Education, Health and Wellbeing, University of Wolverhampton, Millennium City Building, Wulfruna Street, Wolverhampton WV1 1LY, UK; 2 Coventry NIHR CRF Human Metabolism Research Unit, University Hospitals Coventry and Warwickshire NHS Trust, Clifford Bridge Rd, Coventry CV2 2DX, UK; john.hattersley@uhcw.nhs.uk; 3 Warwickshire Institute for the Study of Diabetes, Endocrinology and Metabolism (WISDEM), University Hospitals Coventry and Warwickshire NHS Trust, Coventry CV2 2DX, UK; 4 Centre for Sport, Exercise and Life Sciences, Institute of Health & Wellbeing, Coventry University, Alison Gingell Building, Priory Street, Coventry CV1 5FB, UK; michael.duncan@coventry.ac.uk (M.D.); griffenc@uni.coventry.ac.uk (C.G.); ab9598@coventry.ac.uk (D.R.); ab0289@coventry.ac.uk (J.T.); 5 School of Life Sciences, Faculty of Health and Life Sciences, Coventry University, Alison Gingell Building, Priory Street, Coventry CV1 5FB, UK

**Keywords:** 3D motion analysis, gait analysis, kinetics, joint moment, muscle strength, isokinetic dynamometry

## Abstract

Age-related declines in the musculoskeletal system may place additional demands on the lumbar spine during everyday activities such as walking. This study aimed to investigate age-related differences in the functional demand (FD) of walking on the lumbar spine in older and younger adults. A motion analysis system with integrated force plates was used to acquire kinematic and kinetic data on 12 older (67.3 ± 6.0 years) and 12 younger (24.7 ± 3.1 years) healthy men during walking at a self-selected speed along a 10 m walkway. Isokinetic dynamometry was used to acquire the maximal joint moment capacity of the lumbar spine. The FD of the lumbar spine was calculated as the muscle moment during key phases of the gait cycle (GC) relative to the maximum moment capacity of the lumbar spine. The difference in FD between age groups was not significant (*p* = 0.07) and there were no significant differences between the young group (YG) and older group (OG) for any individual phase in the GC. Despite the lack of statistical significance, the results indicate that a practical difference may exist, as walking was approximately 20% more functionally demanding on the lumbar spine in the OG compared to the YG. Therefore, older adults may employ modified gait strategies to reduce mechanical load whilst walking to fall within the limits of their maximal force-producing capacity in the lumbar spine, which may have implications for injury risk.

## 1. Introduction

Walking, the most common form of physical activity amongst older adults [1], is an important daily task that requires synchronised actions of the musculoskeletal system to function independently. Abnormal gait is predictive of fall incidences and hospitalisation [2,3], but the early identification of gait impairments may improve the effectiveness of interventions and aid in identifying older adults for whom the risk of functional limitations, falls and associated injuries is elevated [4]. However, typical biomechanical markers for identifying gait abnormalities may lack the sensitivity to detect early manifestations of functional decline with normal ageing. For example, a low gait speed (≤0.8 m·s^−1^) is used as the cut-off to determine the severity of sarcopenia once a diagnosis has been confirmed [5]. Single assessments used in isolation may also lack the sensitivity to predict fall risks in older adults [6]. Given the complex and synchronous actions of the whole body, spatiotemporal measures such as walking speed may not always be sufficient to explore the effects of ageing on gait [7], and particularly how demanding the task becomes. This is exemplified by DeVita and Hortobagyi [8] who observed altered kinetics and kinematics with an ageing gait despite performance being similar amongst younger and older adults.

Rather than basing physical function on timed performance, other studies have provided in-depth biomechanical analyses for assessing physical function in older adults [9,10,11]. These studies reported the FD of everyday tasks by normalising joint moments to their maximum capacity. Whilst joint moments provide important information about the total effect of all internal structures (e.g., muscles, ligaments, joint contact forces) that produce a force working across a joint, they do not give a direct measure of how biomechanically demanding a task is for an individual. This is an issue when physiological reserves decline in older adults, as an increase in the biomechanical demand of a task may be masked when internal joint moments are not expressed relative to an individual’s maximal capacity to generate force across a joint. FD, however, provides a measure of the biomechanical challenge of a task and provides an intuitive metric that is easily translated into real-world settings and for comparisons between individuals, as FD is expressed relative to their maximal capacity to produce force across a joint. This is practically relevant and has clinical utility, allowing rehabilitation practitioners and physiotherapists to tailor exercise programmes based on an individual’s requirements (e.g., a patient may have a low strength reserve, a poor movement pattern, or both). To accurately determine FD, the kinematics and contraction type corresponding to the joint used during the movement must be replicated using isokinetic dynamometry. However, this has not been implemented previously as isometric maximal voluntary contractions (MVCs) have been used as the reference for maximal capacity [9,10,11]. For movements such as walking, muscles are constantly changing their role throughout the GC despite their relatively constant activation [12,13,14]. This is demonstrated by the function of muscles transitioning between propulsion and stabilisation, causing their contraction type and angular velocity to change accordingly. Therefore, matching angular velocity and dynamic contraction type during a movement in isokinetic dynamometer test conditions would provide a more realistic FD.

Another gap in the literature is that whilst FD has been applied to the lower limbs [9,10,11], to the authors’ knowledge it has not been applied to the spine. This is likely due to the complexity of the human spine and the fact that lower limb joints are more easily and accurately modelled. Gait is also an activity that is driven by the lower limb musculature and may therefore be seen as more important than the upper body to analyse. However, the trunk muscles are critical in walking and actively contribute to dynamic balance during functional activities [15,16,17,18,19,20]. It should be noted that other studies have focused on similar lumbar loading parameters in healthy adults. These studies [21,22,23,24,25] reported net joint moments or a mechanical power at either L4/5 or L5/S1 in the spine which represent lumbar loading. However, without relating these moments to the maximal force production at the joint (i.e., functional demand), it is difficult to interpret their real-world implication for lumbar loading during everyday activities. Due to changes in trunk kinematics [26] and muscular activation patterns in older age, the mechanical energy demands of the lumbar spine muscles increases [22]. Together with trunk strength reductions [27], older adults may also experience an increase in FD in the lumbar spine during gait. Therefore, understanding the FD placed on the lumbar spine during gait is of clinical importance not only in the presence of pathology, but also in uncovering the effects of ageing. Increasing our knowledge of normal age-related changes in lumbar spine biomechanics is essential for targeted interventions in older adults, as well as for identifying pathological gait patterns.

The aim of this study was to determine the FD of the lumbar spine during normal walking and investigate the difference between older and matched younger adults. The null hypothesis was that there would be no significant difference in the FD of the lumbar spine during walking between the older and younger groups.

## 2. Materials and Methods

The reporting of this prospective observational case-matched study is based on the Strengthening the Reporting of Observational Studies in Epidemiology (STROBE) Statement [28]. Coventry University Ethics Committee approved the study (P70399) on 13 September 2018.

### 2.1. Participants

Following informed written consent, twenty-four community-dwelling volunteers and university staff and students were stratified into the young group (YG; *n* = 12) or older group (OG; *n* = 12). Participants in the YG were matched to participants in the OG based on their physical activity category (assessed using the International Physical Activity Questionnaire—Short Form) [29] and ethnicity. The inclusion criteria were generally healthy males (e.g., free from disease, musculoskeletal injury or functional impairment) aged between 18 and 30 years (YG) or above 60 years (OG). The exclusion criteria were a body mass index (BMI) outside of 18.5–29.9 kg·m^−2^, smokers, the consumption of alcohol on a daily basis, the use of assistive walking equipment, and an existing or past medical history of metabolic diseases, neuromuscular disorders, or musculoskeletal impairments that may affect muscle function. All participants were free living and otherwise healthy individuals, with no history of chronic lumbar conditions, nor undergoing treatment for these conditions. To ensure factors such as pain-related disability and physical activity levels did not confound the results, participants completed the Modified Oswestry Low Back Pain Disability Questionnaire (ODQ-m) [30] and moderate-to-vigorous physical activity (MVPA) was recorded prior to a 3D motion analysis using accelerometery. A full description of this procedure is detailed elsewhere [26]. It should be noted that this study is formed of the secondary analysis of a larger research study.

### 2.2. Three-Dimensional Motion Analysis

Participants wore tight underwear only, allowing markers to be directly attached to the skin, eliminating the marker movement artefacts caused by clothing. Anthropometric measurements and passive retro-reflective marker placement, for all participants, were performed by the same experienced researcher (AD). Thirty-nine markers (14 mm) were attached to anatomical landmarks according to the Plug-in-Gait (PIG) Full Body Marker Model [31]. A Vicon motion capture system comprising 14 infrared cameras (Vero2.2, VMS, Oxford, UK) sampling at 100 Hz and three integrated floor-mounted force plates (AMTI-OPT400600-1K-STT, AMTI, Watertown, MA, USA) sampling at 1000 Hz was used to acquire movement data and ground reaction forces (GRFs). Participants walked barefoot along a 10-metre walkway at a self-selected speed until three successful trials were completed. All gait trials were processed in Vicon Nexus (Version 2.10.1, VMS, Oxford, UK) using standard operations to generate the PIG biomechanical model. The data quality (i.e., unused markers, total gaps, markers labelled and correct labelling) of each trial was inspected. Manual labelling was performed where necessary. Marker trajectory gap filling was initially performed using the Woltring quintic spline filter on up to five samples joined by linear interpolation. Gaps in the marker trajectories of the head, thorax and pelvis segments were filled using the Rigid Body function on up to 25 frames. The remaining gaps of up to 10 frames were interpolated using the Pattern Fill function. Data were then low-pass filtered using a 4th order, zero-lag Butterworth filter with a 10 Hz cut-off frequency. The cut-off frequency was based on the recommendations of Sinclair et al. [32], as 99% of the signal’s power was retained below this point. Each trial was manually truncated to include one full GC (heel strike to heel strike) for each limb. Trials were exported into Vicon Polygon (Version 4.4.5, VMS, Oxford, UK) for further analysis. All outcomes were normalised to one GC (100%) using the linear interpolation of 101 data samples. Each trial consisted of two GCs, and three trials were processed for each participant (i.e., six GCs per participant).

Lumbar spine joint moments (LSJMs) were approximated as the net joint moments (NJMs) between the pelvis and trunk [31]. The full-body PIG model represents the spine as two rigid segments in the pelvis and trunk connected by the lumbar joint. For the purposes of this study, articulation between the pelvis and trunk shall be referred to as the lumbar spine. This model was chosen for this study based on its clinical utility and general representation of the lumbar spine. More detailed musculoskeletal lumbar spine models exist [33,34,35], including a full-body lumbar spine model [36]. However, these may not be practical in clinical settings and more complex models may not generate a positive additional information to computational time balance. This is more pertinent for musculoskeletal models, where the benefit of physiological accuracy may not outweigh the computational time it takes to solve the inverse dynamics and optimisation problems [37].

PIG derives NJMs from the local coordinate frame of the distal segment in the hierarchical kinetic chain [31]. This meant that Vicon Polygon calculated joint moments from an external perspective. The net external moment is predominantly counterbalanced by the net internal moment produced by the muscles. Mathematically, the internal moment is equal and opposite to the external moment [38]. Whilst there is no fundamental difference in adopting an internal or external perspective, the decision should be made based on the researcher’s view of the source of the moments [38]. Given that NJMs were considered to be the result of muscular actions, an internal perspective was adopted, and the moment data were adjusted accordingly. Ensemble averages for LBJMs were calculated in the sagittal plane. Mean peak flexion/extension LBJMs were obtained at the loading response (LR), midstance (MS), terminal stance (TS), pre-swing (PSw), initial swing (ISw) and terminal swing (TSw). The mechanical joint powers of the lumbar spine were calculated from the dot product of the moment and the joint angular velocity vectors. Lumbar spine power was expressed in the sagittal plane to make the results more meaningful physiologically. Joint moments and powers were normalised to the body mass of the participant. Ensemble averages were calculated for flexion/extension moments and the power of the lumbar spine.

### 2.3. Calculating Functional Demand

The FD of the lumbar spine was calculated by dividing the LSJM at the identified instances during the GC by the peak isokinetic MVC moment. The full protocol for obtaining isokinetic moments is described elsewhere [27]. FD was calculated in the sagittal plane only, as trunk isokinetic dynamometry was limited to flexion/extension movements. The mean peak flexion/extension moments of the lumbar spine during LR, MS, TS, PSw, ISw and TSw were identified on the moment–phase curves for each participant. These curves were synchronised with their corresponding power–phase curves to determine the neuromuscular action of the lumbar spine (e.g., extensors concentrically activated) during LR, MS, TS, PSw, ISw and TSw. The moment–phase curves for each participant were then matched up to their corresponding angular velocity–phase curves to determine the angular velocity of the lumbar spine when the identified peak moments occurred. This information was then used to select peak moments from the most appropriate isokinetic condition (i.e., the isokinetic test condition that represented the lumbar spine’s neuromuscular action and movement during the corresponding phase of the GC). For example, if a participant’s lumbar spine extensor muscles were generating power during LR at 30°·s^−1^, a concentric extension isokinetic test at 30°·s^−1^ was sought. It should be noted that 15°·s^−1^ and 30°·s^−1^ were the most typical trunk angular velocities and this did not differ by age. The participant’s peak moment during LR was then divided by their peak isokinetic moment and expressed as a percentage. Therefore, if the participant produced a moment of 1 Nm·kg^-1^ at LR and their peak isokinetic moment was 4 Nm·kg^-1^, the FD would be 25%. If the demand and capacity were equal, the FD would be 100%. For each of the identified phases (LR, MS, TS, PSw, ISw and TSw), individual FD values were calculated and averaged across age groups.

### 2.4. Statistical Analysis

For discrete variables, statistical analyses were performed using SPSS (SPSS^®^ for Windows Version 26.0, IBM Corp., Armonk, NY, USA). A one-way MANOVA was used to compare the difference in FD across all measured GC instances between the OG and YG. Independent-sample *t*-tests were then performed to compare differences at each GC instance between the OG and YG. Age-group differences in kinetic waveforms were compared using Statistical Parametric Mapping (SPM) two-tailed independent *t*-tests [39], which were performed in MATLAB R2019a (v. 9.6.0) and implemented using the open source one-dimensional Statistical Parametric Mapping code (spm1D-package, version 0.4.3, http://spm1d.org/index.html, accessed on 15 July 2020). This test was performed to investigate whether there were phase-specific (i.e., continuous rather than discrete) differences between the age groups. The alpha level was set to 5% for all statistical tests and effect sizes were calculated where appropriate (i.e., Cohen’s *d* for *t*-tests and η_p_^2^ for MANOVA). All data were normally distributed, as assessed by Shapiro–Wilk tests (*p* > 0.05). Where the assumption of the homogeneity of variances was violated, as assessed by Levene’s Test of Equality of Variances (*p* < 0.05), the Welch–Satterthwaite correction was used. Data are presented as means with standard deviations (mean ± SD) unless otherwise stated. Graphical presentations were performed using GraphPad Prism (Version 8.3.1, San Diego, CA, USA), apart from for the SPM output.

## 3. Results

No significant differences (*p* > 0.05) in participant characteristics, except for age, were observed. The spatiotemporal gait parameters between the YG and OG were also comparable (*p* > 0.05) (Table 1).

### 3.1. Lumbar Spine Moments and Power

There were significant age differences in terms of peak extension LBJM during MS (*t*(22) = 2.28, *p* = 0.032) and peak flexion LBJM during TSw (*t*(22) = −2.16, *p* = 0.042). The peak extension moments were on average 54.8% greater in the YG (0.96 ± 0.45 Nm·kg^−1^) compared to the OG (0.62 ± 0.24 Nm·kg^−1^) during MS, whereas during TSw the OG produced greater peak flexion moments (1.05 ± 0.37 Nm·kg^−1^) than the YG (0.74 ± 0.34 Nm·kg^−1^) (Table 2).

**Figure 1:**
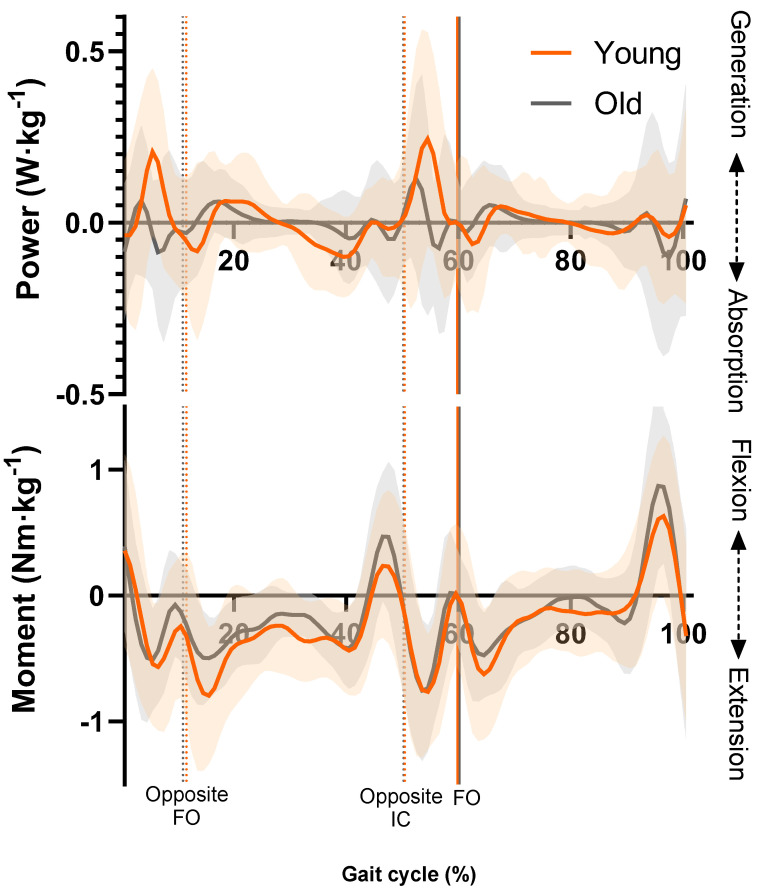
Ensemble averages for lumbar spine joint power and moments in the sagittal plane. Orange line = YG mean, grey line = OG mean, orange shaded area = YG SD, grey shaded area = OG SD. FO = foot off, IC = initial contact.

**Figure A1:**
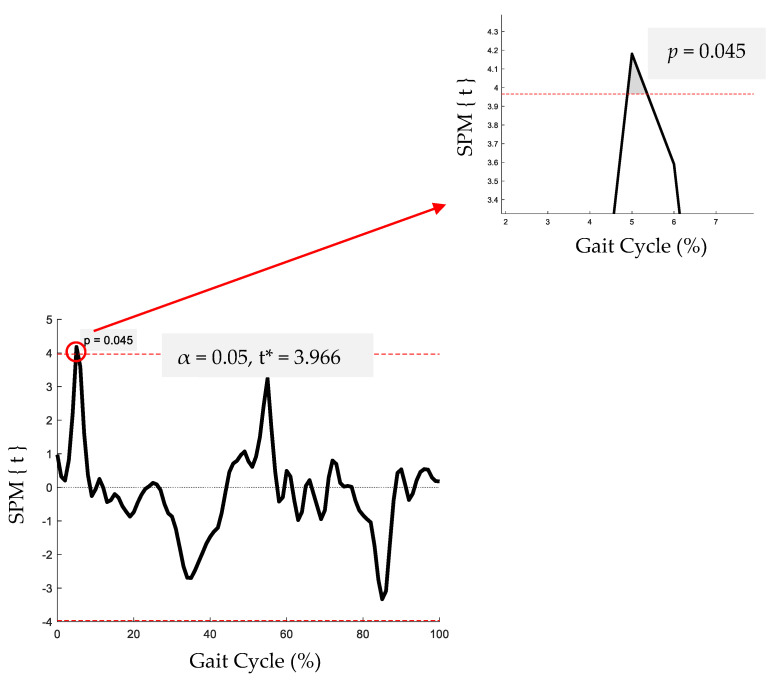
Statistical Parametric Mapping (SPM) output for lumbar spine joint power. The horizontal red dashed lines represent the critical t* based on α = 0.05 and random field theory calculations of residual smoothness. t* is the critical threshold.

The YG displayed significantly greater lumbar spine extensor joint power than the OG during LR (Figure 1). More precisely, the SPM showed that the YG exhibited power generation in their extensor muscles whilst the OG exhibited power absorption during this period. This was shown by a supra-threshold cluster (4.9–5.4%) exceeding the critical threshold of *t*(22) = 3.966, *p* = 0.045 (Figure A1). No phase-specific differences between age groups were found for the LBJMs.

### 3.2. Functional Demand

**Figure 2:**
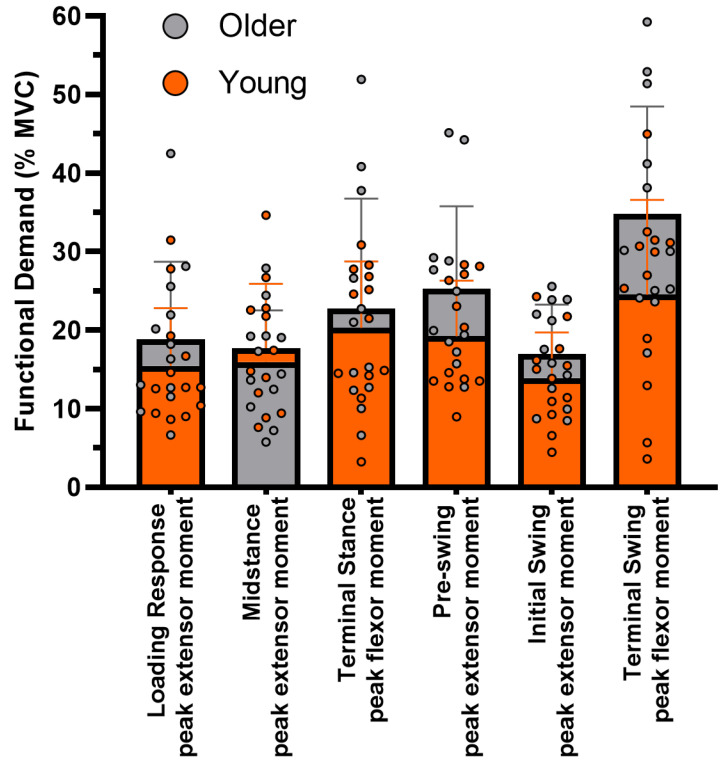
Functional demand of the lumbar spine at key instances during the gait cycle. Bars and error bars represent mean ± SD. Orange bars represent the YG, grey bars represent the OG.

The omnibus test comprising all the GC instances measured approached statistical significance (F(6, 17) = 2.40, *p* = 0.073; Wilk’s ∆ = 0.54, η_p_^2^ = 0.46), meaning that the overall difference in FD between age groups, whilst not statistically significant, was large. There were no significant differences in FD between the YG and OG for any individual phase during the GC. The FD was generally higher in the OG, except during MS, where the YG’s mean peak extensor moment was closer to their MVC. The difference in FD at TSw was greatest between groups and approached statistical significance (*t*(22) = −1.97, *p* = 0.062). The FD during TSw was on average 42% greater in the OG (34.8 ± 13.6%) compared to the YG (24.5 ± 12.1%). Across the phases shown in Figure 2, the mean FD in the lumbar spine was approximately 20% greater in the OG.

## 4. Discussion

Age-related changes in spinal biomechanics are not fully understood. As the trunk muscles play a crucial role in mobility and postural support during walking, it is important to understand normal age-related changes in how functionally demanding gait is on the lumbar spine. The current study addressed this gap in the literature by investigating differences in the FD of the lumbar spine during walking between healthy younger and older adults. Previously [9,10,11], the denominator in calculating FD has been an isometric MVC, which is not reflective of dynamic tasks such as walking and artificially increases the FD. The current study is the first to determine the age effect on FD in the lumbar spine using a biomechanically applicable metric that accounted for neuromuscular action and angular velocity. This is important because it provides a more realistic understanding of the biomechanical challenge posed by walking in the lumbar spine in older adults. Whilst the results showed no significant differences between the older and younger groups, the overall difference in FD across the GC was large and may be clinically or practically relevant, indicating that walking may be a more challenging task for the lumbar spine in older people.

**Figure A2:**
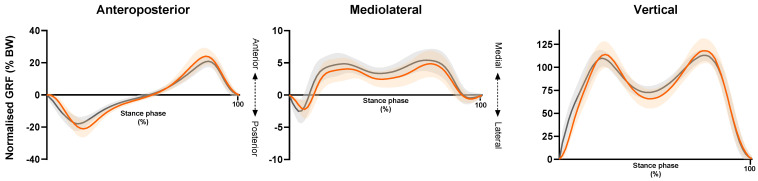
Ensemble averages for ground reaction forces. Orange line = YG mean, grey line = OG mean, orange shaded area = YG SD, grey shaded area = OG SD.

We have previously shown that the OG in this study demonstrated a greater anterior trunk tilt compared to the YG [26]. Additional demands may be placed on the lumbar spine musculature in older adults as a consequence of flexed postures [24]. Greater internal extension moments generated by the paravertebral muscles are required to balance the external flexion moment about the lumbar spine caused by the anterior shift in the centre of mass (COM) of the upper body [40]. However, this was not seen in the current results. Generally, the OG produced lower extension moments at key instances during the GC and the moment waveform patterns and GRFs (Figure A2) were similar between the groups. As previously reported for this cohort [23], the OG had a lower strength reserve; this may have masked differences in FD compared to the YG, as capacity and demand were both reduced in the OG. Albeit not significant, the OG generally operated nearer their maximal capacity to generate and absorb flexion/extension moments, particularly near the end of the GC. This was highlighted by the significantly greater flexion moment absorbed by the OG during TSw compared to that of the YG. Whilst this eccentric activity in the trunk flexors may be a stability mechanism to reduce the posterior translation of the body’s COM over its base of support in preparation for impact at initial contact, it is likely that the resulting motion of extension aids in the forward progression of the body’s COM [41]. Indeed, a lumbar spine extension may also act as a stabilising mechanism for hip extensor activity, particularly during LR, when the hip extensors control a large external flexor moment [41].

The OG’s altered neuromuscular control of their lumbar spine may have also led to differences in the FD being undetectable. The OG fluctuated between extensor power generation and absorption, whilst the YG predominantly generated extensor power during LR. Peak power generation was also smaller in the OG. This disparity was replicated in the second period of double limb support during PSw. Another study supports this finding that the locomotor function of the trunk is altered in older age during gait [22]. However, unlike the current study, McGibbon and Krebs [22] report moments without relating them to individuals’ maximal force output. This demonstrates the uniqueness of current study, in that FD considers both the reduction in physiological strength reserves and the internal forces driving the motion or required to withstand external forces. Furthermore, there are no studies reporting moments relative to maximal force capacity to which the current study can be specifically compared. This lack of direct comparison further emphasises the novelty of the current study. It is possible that younger adults contract their lumbar spine muscles concentrically during double limb support to adopt a pelvic-leading gait strategy. This is more efficient than a trunk-leading strategy, seen in older adults, which results in a greater mechanical energy expenditure of the lumbar spine musculature due to its eccentric activation [22]. Consistent with the current findings, McGibbon and Krebs [22] found that older adults have a greater reliance on the eccentric control of their lumbar paravertebral muscles to mediate energy transfer during double limb support phases. Given that maximal force production is greater for eccentric actions than concentric, this may explain why the FD was somewhat greater during the GC for the OG. This mechanism, however, cannot be fully supported by the current results as the difference in FD between the age groups was not significant and may have occurred due to chance. Lower power generation in the lumbar spine may be indicative of the OG reducing concentric muscle activity to minimise the energy transferred proximally to the trunk during periods of less stability such as single limb support [22]. The current results exemplify this as the YG generated significantly greater peak extensor moments during MS compared to the OG. This is supported by EMG studies showing lower muscle activity in the erector spinae and psoas muscle groups of older adults compared to younger adults during walking [17,42]. A more conservative gait strategy which solicits less activation of the lumbar paravertebral muscles (i.e., lower joint moments produced by muscles to control external moments) provides a plausible mechanism explaining why older adults are able to somewhat attenuate increases in FD despite a lower capacity to develop force in these muscles.

### 4.1. Clinical and Practical Applications

The upper body is generally overlooked in clinical gait analysis compared to the lower limbs. Whilst not unequivocal, the data from the present study indicate that the lumbar spine should be considered during gait to inform the design of more effective strategies for preserving physical function in older age. Understanding age-related changes in the FD of the lumbar spine could assist clinical decision making and rehabilitation programmes with regard to implementing age-specific resistance or motor skill training. FD, as a composite measure of capacity and demand, is useful in determining physical capability and monitoring declines in the physical function of older adults. It also provides an intuitive metric that is easily compared to other individuals and groups of interest.

The current findings indicate that during low-impact everyday activities such as walking, the lumbar spine muscles are challenged somewhat more in older age. This finding could support a targeted approach by identifying whether functional capacity (i.e., strength) or demand (i.e., moments exerted during a task) should be prioritised (through resistance and motor skills training, respectively) to improve physical function in older age. Routinely analysing spinal mechanics will also increase the evidence base and establish normative data, which could be used to identify abnormal patterns that may not be apparent in the lower limbs. Furthermore, the clinical utility of the full-body PIG model is acceptable where a balance of accuracy and practicality is needed, such as in clinical settings. The use of isokinetic dynamometry, however, is still not routine in clinical settings. Given the advancements in this technology over recent years, isokinetic dynamometry is a relatively practical and reliable solution for determining the force-producing capacity of muscles in the lumbar spine [43,44], as well as other joints in the body. Consensus on a standardised approach is needed to establish reference values for trunk strength in pathological groups such as lower back pain patients [45,46], as well as generally healthy [47] and athletic groups [43].

### 4.2. Limitations

There are limitations to this study that should be acknowledged. PIG can estimate lumbar spine kinetics; the spine is represented by the two rigid segments of the pelvis and the trunk, connected by the lumbar joint [48,49]. This model is similar to modelling approaches previously used to determine lumbar spine moments, wherein the trunk is considered a single rigid segment that is attached distally to the pelvis at a single lumbosacral joint [24]. Whilst this reduces the complex articulations of the lumbar spine to one joint; it provides a gross understanding of the biomechanics in this region. Greater accuracy at individual vertebral levels may be elicited with more complex spinal models. However, they may also increase measurement errors as the size of functional spinal units is small and limits our ability to position three non-colinear markers on the skin overlying multiple functional spinal units [50]. Increases in data collection and computational requirements may also reduce the clinical utility of more detailed approaches [51]. Confidence in the current results is high nonetheless, as the moments of the OG and YG were comparable to those produced in previous studies using a similar modelling approach [23,24], as well as ones using more complex musculoskeletal models [52]. It should also be noted that the isokinetic dynamometer protocol approximated the lumbosacral region of the spine. Therefore, both measurement tools were representative of a more global approach to determining the FD of the lumbar spine. It would be practically unfeasible to use isokinetic dynamometry at individual vertebral levels with any degree of accuracy. Our sample comprised healthy active younger and older men. Given that trunk biomechanics are modified by disease, pain and physical impairment [49,53], caution should be taken when generalising the present study’s findings to populations other than healthy men. This study was also specific to walking and age-related differences in FD may be more apparent for more challenging movements such as stair negotiation and sit-to-stand. Caution should also be taken when generalising these findings to healthy older populations given the relatively small sample size.

Finally, it was not possible to calculate the FD for the lateral flexion and axial rotation of the lumbar spine in the current study. Therefore, it is unknown whether walking was more functionally demanding for the OG in the coronal and transverse planes compared to the YG. Obtaining isokinetic strength data on the lateral flexion and axial rotation of the trunk may reveal useful findings about the FD of walking in other planes. It should also be noted that FD is a relative measure of an individual’s maximal capacity to produce muscular force. Whilst it is an intuitive measure and provides useful information, it does not account for the fatigue-resistant capabilities of the muscle group being assessed. Understanding the FD of the lumbar spine during gait across longer distances or time periods may reveal other age-related mechanisms associated with neuromuscular fatigue. Since older adults are arguably more susceptible to the detrimental effects of muscle fatigue than younger adults [54,55,56], it is likely that the musculature in their lumbar spine would not respond as well to the demand for prolonged force production. This may have greater consequences in older populations, limiting their ability to perform longer-duration walking.

## 5. Conclusions

This study offers new insight into the effect of age on the biomechanical function of the lumbar spine during gait. FD has previously been calculated using static MVCs as the reference for maximal force production capacity, which is not reflective of human movement in everyday life. In the present study, FD was calculated using biomechanically relevant metrics that replicate the neuromuscular function of the lumbar spine during walking. The key finding was that older age appears to somewhat increase the FD on the lumbar spine during walking. Whilst differences with the YG were not statistically significant, this may be due to the OG modifying their gait strategy to reduce demand during the task to closer to their force-producing capacity. Future research should focus on a range of age groups and diseased populations to further our understanding of how functionally demanding everyday tasks become in older age and for people living with disabilities. Other activities of daily living (e.g., stair negotiation) warrant further research to identify which tasks present the greatest challenge to older adults. Also, longer exposures to routine activities such as walking longer distances may reveal more nuanced age effects on FD.

## Figures and Tables

**Table 1:** Participant characteristics and spatiotemporal parameters during normal gait (mean ± SD) for the younger and older groups.

Parameter	Younger Group (*n* = 12)	Older Group (*n* = 12)	Independent *t*-Test	Cohen’s d
**Participant characteristics**				
*Age (years)*	*24.7 ± 3.1*	*67.3 ± 6.0*	*t(22) =* −*21.8, p < 0.001*	*8.92*
Height (m)	1.78 ± 0.1	1.74 ± 0.1	*t*(22) = 1.2, *p* = 0.23	0.40
Mass (kg)	76.4 ± 11.2	79.2 ± 10.8	*t*(22) = -0.6, *p* = 0.55	0.25
BMI (kg.m^−2^)	24.1 ± 2.2	26.0 ± 2.7	*t*(22) = −1.9, *p* = 0.07	0.77
ODQ-m (%)	2.2 ± 2.3	2.2 ± 3.5	*t*(22) = 0.0, *p* = 1.00	0.00
MVPA (hours per day)	6.6 ± 1.4	6.3 ± 1.5	*t*(22) = 0.5, *p* = 0.60	0.21
**Spatiotemporal data**				
Walking Speed (m·s^−1^)	1.45 ± 0.19	1.33 ± 0.16	*t*(22) = 1.70, *p* = 0.10	0.69
Cadence (steps·min^−1^)	113.9 ± 5.8	113.0 ± 7.2	*t*(22) = 0.34, *p* = 0.74	0.14
Step Length (m)	0.76 ± 0.08	0.71 ± 0.06	*t*(22) = 1.85, *p* = 0.08	0.76
Normalised Step Length	0.43 ± 0.05	0.41 ± 0.03	*t*(19.7) = 1.43, *p* = 0.17	0.59
Step Width (m)	0.14 ± 0.03	0.16 ± 0.03	*t*(22) = −1.22, *p* = 0.23	0.50

Note: italics denote significant age effect; MVPA = moderate-to-vigorous physical activity, BMI = body mass index, ODQ-m = Modified Oswestry Low Back Pain Disability Questionnaire; Normalised Step Length was relative to height in metres.

**Table 2:** Lumbar spine joint moment peaks (mean ± SD) for the younger and older groups during normal gait.

Neuromuscular Action and Gait Cycle Phase	Younger Group (Nm·kg^−1^)	Older Group (Nm·kg^−1^)	Independent *t*-Test	Cohen’s d
Extensor moment—LR	0.78 ± 0.39	0.74 ± 0.26	*t*(22) = 0.31, *p* = 0.76	0.13
Extensor moment—MS *	0.96 ± 0.45	0.62 ± 0.24	*t*(22) = 2.28, *p* = 0.032	0.93
Flexor moment—TS	0.63 ± 0.27	0.70 ± 0.41	*t*(22) = −0.50, *p* = 0.62	0.20
Extensor moment—PSw	0.97 ± 0.34	0.99 ± 0.25	*t*(22) = −0.15, *p* = 0.89	0.06
Extensor moment—ISw	0.73 ± 0.29	0.63 ± 0.20	*t*(22) = 1.01, *p* = 0.33	0.41
Flexor moment—TSw *	0.74 ± 0.34	1.05 ± 0.37	*t*(22) = −2.16, *p* = 0.042	0.88

* = significant age effect; LR = loading response, MS = midstance, TS = terminal stance, PSw = pre-swing, ISw = initial swing, TSw = terminal swing.

## Data Availability

The data presented in this study are not publicly available due to privacy or ethical restrictions.
